# Association Between Biopsy PD-L1 Combined Positive Score and Pathological Upgrading at Radical Prostatectomy in Grade Group 1 Prostate Cancer: A Retrospective Study

**DOI:** 10.3390/ijms27177878

**Published:** 2026-09-03

**Authors:** Ludovica Pepe, Valeria Zuccalà, Ida Rizzuto, Walter Giuseppe Giordano, Mariagiovanna Ballato, Pietro Tralongo, Gabriele Ricciardi, Vincenzo Cianci, Marta Rossanese, Giuseppe Iatì, Silvana Parisi, Francesco Pierconti, Antonio Ieni, Guido Fadda, Pietro Pepe, Maurizio Martini, Vincenzo Fiorentino

**Affiliations:** 1 Anatomic Pathology Unit, Department of Human Pathology in Adult and Developmental Age “Gaetano Barresi”, University of Messina, 98125 Messina, Italy; ludovica.pepe@studenti.unime.it (L.P.);; 2 PhD Program in Translational Molecular Medicine and Surgery, Department of Biomedical, Dental, Morphological and Functional Imaging Sciences, University of Messina, 98125 Messina, Italy; mariagiovannaballato96@gmail.com (M.B.); pietrotralongo@gmail.com (P.T.); gricciardi1998@gmail.com (G.R.); 3 Section of Legal Medicine, Department of Biomedical, Dental, Morphological and Functional Imaging Sciences, University of Messina, 98125 Messina, Italy; 4 Division of Urology, Department of Human Pathology in Adult and Developmental Age “Gaetano Barresi”, University of Messina, 98125 Messina, Italy; 5 Radiation Oncology Unit, Department of Biomedical, Dental, Morphological and Functional Imaging Sciences, University of Messina, Via Consolare Valeria 1, 98124 Messina, Italy; 6 Department of Clinical and Experimental Medicine (DIMED), University of Messina, Via Consolare Valeria 1, 98124 Messina, Italy; 7 Division of Anatomic Pathology and Histology, Fondazione Policlinico Universitario “Agostino Gemelli” IRCCS, Università Cattolica del Sacro Cuore, Largo Francesco Vito 1, 00168 Rome, Italy; 8 Urology Unit, Cannizzaro Hospital, 95126 Catania, Italy

**Keywords:** PD-L1, prostate cancer, Grade Group 1, combined positive score, pathological upgrading, prostate biopsy

## Abstract

Programmed death-ligand 1 (PD-L1) expression in Grade Group 1 (GG1) prostate carcinoma biopsies has not been established as a marker of pathological upgrading at radical prostatectomy (RP). This two-center retrospective case–control study included 172 men with GG1 carcinoma in the biopsy closest to RP: all 86 patients upgraded to GG2 and 86 randomly selected non-upgraded controls from 126 eligible GG1 patients. PD-L1 was assessed with the SP263 clone using the combined positive score (CPS), with CPS ≥ 1 predefined before outcome comparison. CPS ≥ 1 occurred in 36/86 upgraded cases (41.9%) and 18/86 controls (20.9%; unadjusted odds ratio [OR], 2.72; 95% confidence interval [CI], 1.39–5.33; *p* = 0.005). After adjustment for serum prostate-specific antigen, positive-core count, Prostate Imaging Reporting and Data System category, and center, CPS ≥ 1 remained associated with upgrading (adjusted OR, 2.91; 95% CI, 1.47–5.97; *p* = 0.003). A Firth bias-reduced sensitivity model including digital rectal examination yielded a similar CPS estimate. Biopsy CPS ≥ 1 was associated with biopsy–RP grade discordance, but the study does not establish an optimal cutoff, predictive performance, or clinical utility. Prospective multicenter validation, including active-surveillance outcomes, is required.

## 1. Introduction

Prostate carcinoma (PCa) is the second most commonly diagnosed malignancy in men worldwide and the most frequently diagnosed cancer among men in Europe [1,2]. Its clinical behavior ranges from indolent localized disease to aggressive metastatic cancer [3]. Reliable risk stratification is therefore essential to maintain oncological safety while avoiding unnecessary treatment. Contemporary assessment combines serum prostate-specific antigen (PSA), clinical examination and staging, multiparametric magnetic resonance imaging (mpMRI), and histopathological grading of needle-biopsy specimens [4,5].

Histological grade, reported using the Gleason system and the International Society of Urological Pathology (ISUP) Grade Group (GG) classification, remains a major determinant of prognosis and treatment selection [5]. Biopsy grade, however, does not always correspond to the grade in the radical prostatectomy (RP) specimen. Upgrading is reported in approximately 25–40% of patients, depending on the study population and biopsy approach [6,7,8,9]. Combining mpMRI-targeted biopsy with systematic or perilesional sampling improves detection of clinically significant disease and biopsy–RP concordance but cannot eliminate sampling error or fully account for intratumoral heterogeneity [4,7,8].

Immune checkpoint blockade has improved outcomes in several malignancies, yet PCa generally exhibits an immune-cold microenvironment [10,11]. In unselected metastatic castration-resistant PCa, responses to pembrolizumab are uncommon, and the addition of pembrolizumab to docetaxel has not improved survival [12,13]. Clinical benefit is more consistently observed in molecularly selected tumors, particularly those with microsatellite instability or mismatch repair deficiency [14].

The programmed cell death protein 1 (PD-1)/programmed death-ligand 1 (PD-L1) pathway contributes to tumor–immune interactions and immune escape [15,16], but the clinicopathological significance of PD-L1 expression in PCa remains uncertain. Published immunohistochemical studies have ranged from sparse expression in untreated primary tumors to associations with higher grade, aggressive pathological features, or biochemical recurrence [17,18,19,20,21,22,23,24,25,26]. Variations in cohort composition, specimen type, pre-analytical handling, antibody clone and platform, scoring method, and positivity threshold probably account for some of this heterogeneity.

Active surveillance (AS) is widely recommended for appropriately selected men with biopsy GG1 (Gleason score [GS] 3 + 3) PCa [4]. Long-term prospective evidence supports its oncological safety, with a 15-year cumulative incidence of metastasis of 2.7% in a large surveillance cohort [27]. Repeat biopsy, with MRI-targeted sampling, when indicated, remains important for detecting clinically significant reclassification during surveillance [28]. Nonetheless, a GG1 biopsy may underestimate the true extent or grade of disease. Multifocality, sampling limitations, and intratumoral heterogeneity can leave higher-grade foci unsampled and result in upgrading at RP [6,8,9]. Tissue-based and genomic assays may refine risk assessment in selected patients, although current recommendations do not support routine testing in all men with localized disease [29].

The combined positive score (CPS) provides a semiquantitative measure of PD-L1 expression in tumor cells and associated mononuclear inflammatory cells. A threshold of CPS ≥ 1 has been used in PCa trials and pathological series [12,19,20,30,31], but it has not been validated in localized GG1 disease [22]. Because lymphocytes and macrophages contribute to the numerator, CPS may reflect local inflammatory activity as well as PD-L1 expression by neoplastic cells. It is not known whether PD-L1 expression in a GG1 biopsy is associated with unsuspected higher-grade disease in the subsequent RP specimen.

We therefore evaluated whether CPS in the preoperative GG1 biopsy closest to RP was associated with upgrading to GG2 at RP.

## 2. Results

Between January 2020 and January 2025, 212 consecutive men met the eligibility criteria at the two participating centers. All had GS 3 + 3 (ISUP GG1) in the biopsy closest to RP and sufficient residual tissue for PD-L1 immunohistochemistry. Institutional RP reports classified 86 patients (40.6%) as GG2 and 126 as GG1; no patient had GG3 or higher disease.

**Figure 1:**
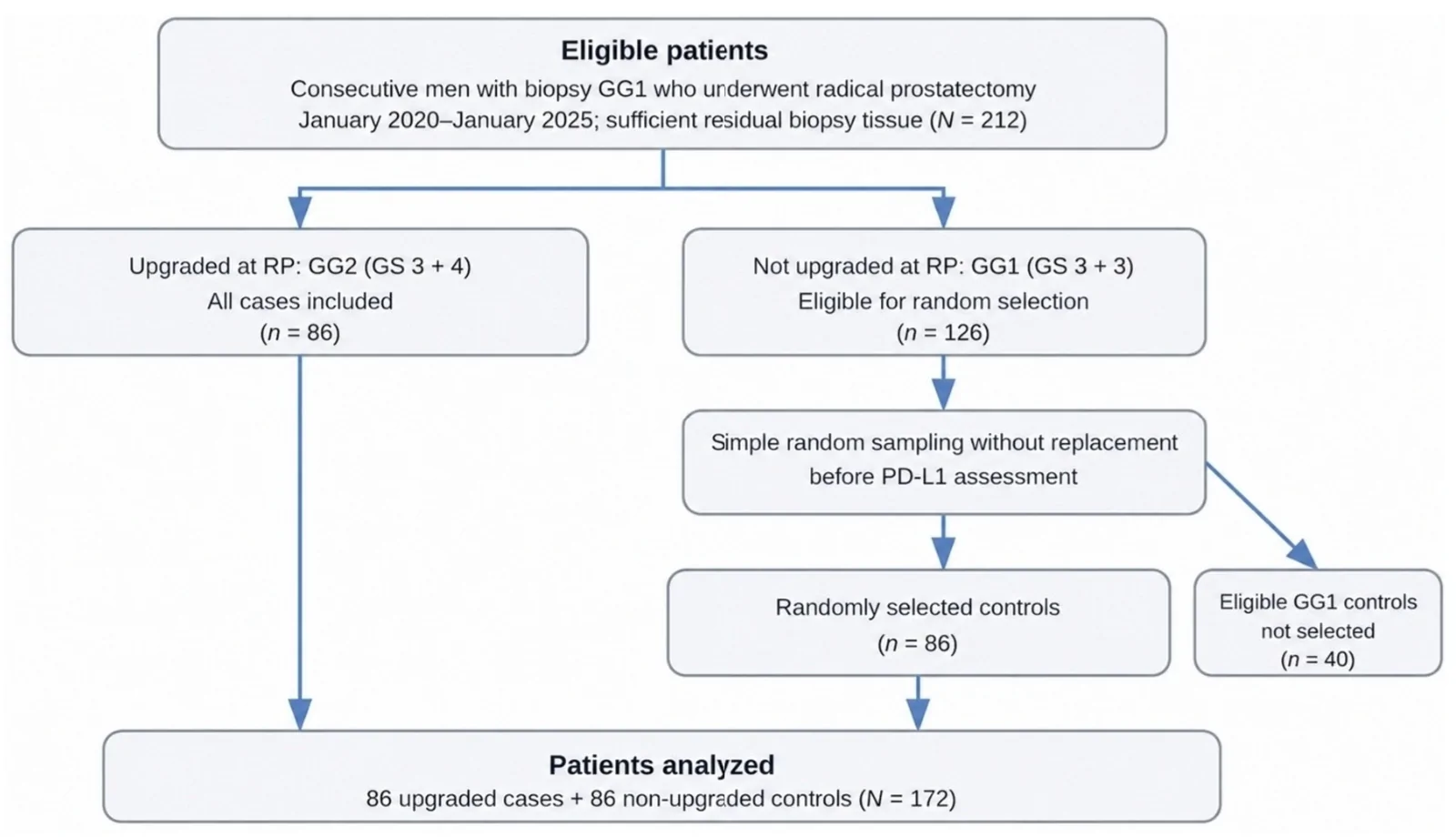
Study flow and random selection of controls. Among 212 eligible men, institutional RP reports classified 86 as GG2 and 126 as GG1; none had GG3 or higher disease. All 86 GG2 patients were included as cases. Of the 126 GG1 patients, 86 were randomly selected as controls, and 40 were not selected. Blinded central review confirmed the institutional classification of all 172 RP specimens. Abbreviations: *N*, total number of patients at the relevant stage; *n*, number of patients in each subgroup; GG, grade Group; GS, Gleason score; PD-L1, programmed death-ligand 1; RP, radical prostatectomy.

All 86 patients classified as GG2 were included as cases. Among the 126 patients who remained GG1 at RP, 86 controls were selected by simple random sampling before PD-L1 assessment and without reference to clinical, pathological, biopsy-related, or institutional characteristics. No matching or stratification was used in the primary design. The remaining 40 eligible GG1 patients were not selected. The final analytic cohort therefore comprised 172 patients (Table 1; Figure 1).

Although none of the descriptive comparisons in Table 1 reached *p* < 0.05, random selection does not guarantee covariate balance. Within the primary analytic sample, the largest absolute standardized mean differences were 0.26 for abnormal DRE, 0.18 for age, 0.16 for positive-core count, and 0.16 for mixed tumor location. These values describe residual imbalance within the selected analytic sample (Table S4).

The median interval from the biopsy used for PD-L1 assessment to RP was 86 days (interquartile range [IQR], 62–114) in upgraded cases and 78 days (IQR, 60–111) in controls.

CPS ≥ 1 was detected in 36 of 86 upgraded cases (41.9%) and 18 of 86 controls (20.9%). This corresponded to an unadjusted odds ratio (OR) for upgrading of 2.72 (95% confidence interval [CI], 1.39–5.33; Fisher’s exact test, *p* = 0.005; Table 2).

CPS values were right-skewed. The median CPS was 0 [IQR, 0–0; range, 0–20] in controls and 0 [IQR, 0–1; range, 0–20] in upgraded cases. In exploratory categories of 0, 1–5, and >5, the corresponding counts were 68, 16, and 2 among controls and 50, 32, and 4 among upgraded cases. In an adjusted ordered-category association model, the OR was 2.44 per category increase (95% CI, 1.35–4.62; *p* = 0.004), whereas CPS modeled linearly per one-point increase was not significantly associated with upgrading (adjusted OR, 1.09; 95% CI, 0.97–1.27; *p* = 0.208; Table S1). Because the ordered model imposes a common log-odds increment across categories and only six patients had CPS > 5, it should not be interpreted as demonstrating a dose–response. These analyses were not used to select or optimize a cutoff.

On central review, all biopsies used for PD-L1 assessment remained GG1 and contained no Gleason pattern 4, and all institutional GG1/GG2 classifications of the 172 RP specimens were confirmed. No biopsy showed cribriform morphology or intraductal carcinoma. In RP specimens, cribriform morphology was identified in 9 patients (5.2%), intraductal carcinoma in none, and pT3a disease in 10 (5.8%); no pT3b tumors were present. Before consensus review, Cohen’s κ was 0.92 for final RP Grade Group classification (GG1 vs. GG2) and 0.83 for binary biopsy CPS classification (CPS < 1 vs. CPS ≥ 1).

Because abnormal DRE was present in only nine patients, DRE was removed from the primary multivariable model and evaluated in sensitivity analyses. In the primary model, CPS ≥ 1 remained associated with upgrading after adjustment for PSA, positive-core count, PI-RADS category, and center (adjusted OR, 2.91; profile-likelihood 95% CI, 1.47–5.97; *p* = 0.003; Table 3). PI-RADS was modeled as an ordinal variable, complete covariate data were available for all patients, and all variance inflation factors were ≤1.06.

Sensitivity analyses yielded comparable CPS estimates when DRE was added to the conventional model (adjusted OR, 2.91; 95% CI, 1.46–5.99), biopsy type was added (3.14; 95% CI, 1.56–6.58), age was added (2.94; 95% CI, 1.48–6.04), positive-core count was omitted (2.98; 95% CI, 1.51–6.08), or PI-RADS was modeled categorically (2.89; 95% CI, 1.45–5.95). Firth bias-reduced logistic regression including DRE produced an adjusted CPS OR of 2.77 (95% CI, 1.41–5.61; penalized likelihood-ratio *p* = 0.003). The abnormal-DRE coefficient remained imprecise (OR, 2.93; 95% CI, 0.72–16.59; *p* = 0.137). Influence analyses did not identify individual observations that materially altered the CPS estimate (Tables S2 and S3).

**Figure 2:**
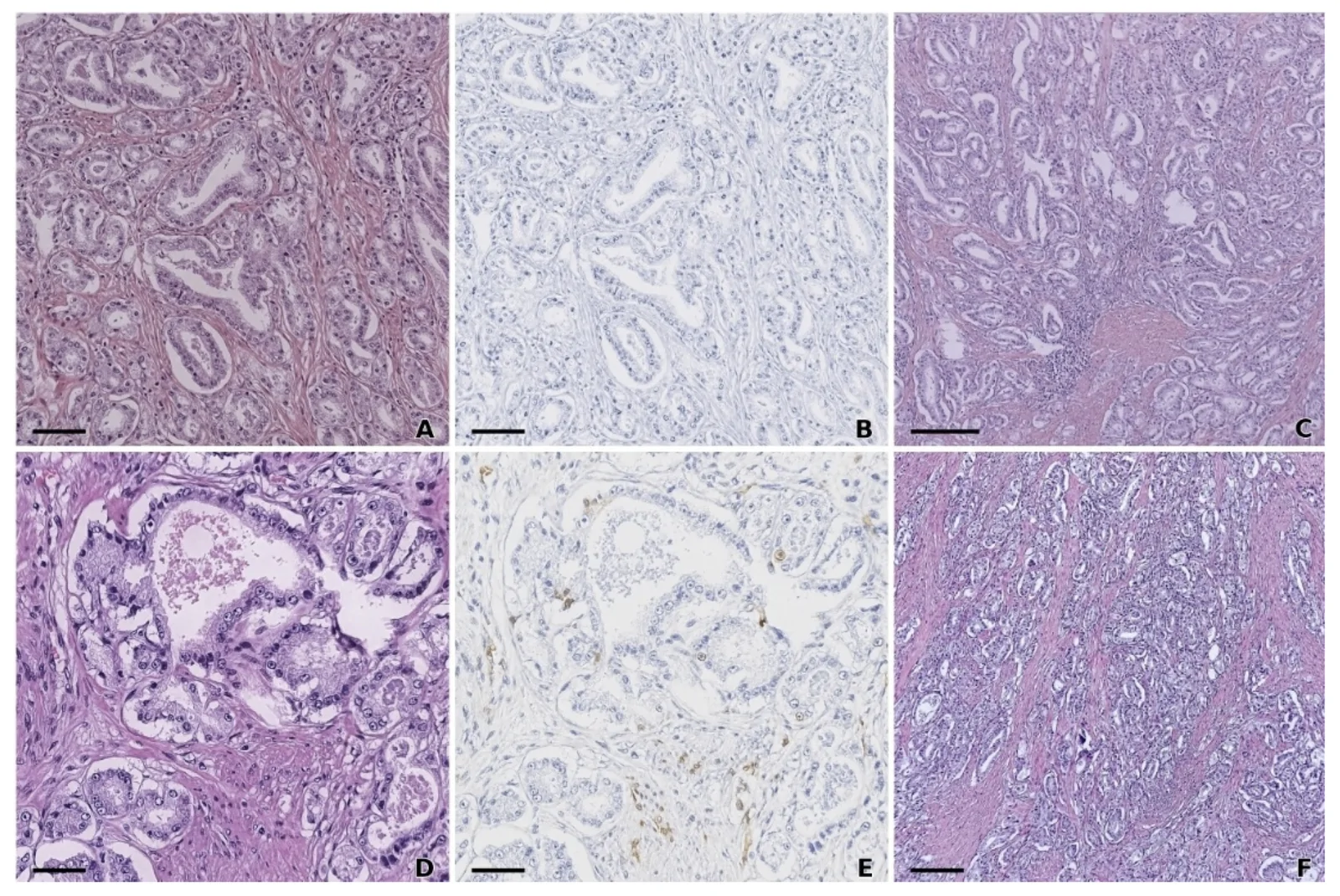
Representative paired biopsy and RP findings. Panels (**A**–**C**) show findings from a patient without upgrading: (**A**) hematoxylin and eosin (H&E)-stained needle-core biopsy showing prostatic acinar adenocarcinoma, Grade Group 1 (Gleason score [GS] 3 + 3 = 6); (**B**) PD-L1 immunohistochemistry performed on a consecutive section from the corresponding tumor-bearing region, showing negative staining (combined positive score [CPS] < 1); and (**C**) matched RP specimen confirming Grade Group 1 acinar adenocarcinoma (GS 3 + 3 = 6). Panels (**D**–**F**) show findings from a patient with upgrading: (**D**) H&E-stained pre-radical-prostatectomy needle-core biopsy showing infiltrative glands diagnostic of prostatic acinar adenocarcinoma, Grade Group 1 (GS 3 + 3 = 6); (**E**) PD-L1 immunohistochemistry performed on a consecutive section from the corresponding tumor-bearing region, showing focal immunoreactivity in scattered tumor-associated mononuclear inflammatory cells (CPS ≥ 1); and (**F**) matched RP specimen showing Grade Group 2 acinar adenocarcinoma (GS 3 + 4 = 7). Images in panels (**A**,**B**,**D**,**E**) were acquired using a 20× objective, whereas those in panels (**C**,**F**) were acquired using a 10× objective. Scale bars represent 50 μm in panels (**A**,**B**,**D**,**E**) and 100 μm in panels (**C**,**F**). Abbreviations: CPS, combined positive score; GS, Gleason score; H&E, hematoxylin and eosin; PD-L1, programmed death-ligand 1.

Representative paired biopsy and RP findings are shown in Figure 2. Serial biopsy sections illustrate the same tumor-bearing area, first on hematoxylin and eosin (H&E) staining and then with the corresponding pattern of PD-L1 immunoreactivity, whereas the matched RP sections document the final GG.

## 3. Discussion

In this two-center case–control study of men with GG1 PCa on preoperative biopsy, CPS ≥ 1 was associated with nearly threefold higher adjusted odds of upgrading to GG2 at RP. The association remained consistent in Firth bias-reduced sensitivity analysis and across alternative covariate specifications. Biopsy CPS ≥ 1 was associated with biopsy–RP grade discordance, but its predictive performance and clinical utility were not evaluated.

Studies examining the relationship between PD-L1 expression, GG, and other adverse pathological features in PCa have yielded inconsistent results [17,18,19,20,21,23,24,25,26]. Published cohorts have reported either little PD-L1 expression in untreated primary PCa or associations with aggressive features, macrophage-rich microenvironments, or biochemical recurrence [23,24,25,26]. In a previous multi-institutional study, which included 120 biopsies from 52 patients, CPS was associated with both GG and cribriform morphology [20]. Differences in specimen type, assay conditions, scoring methods, and positivity thresholds limit direct comparisons across studies [22].

These observations should not be interpreted in the context of immunotherapy selection. Although checkpoint inhibitors are effective in several malignancies [32,33,34,35], their activity in unselected PCa remains limited [12,13,30,31] and is more evident in molecularly defined subsets [14]. CPS ≥ 1 has been used to define PD-L1-positive subgroups in prostate cancer trials [12,30,31], but the endpoint in this study was grade discordance between biopsy and RP, not response to immune checkpoint blockade.

The CPS ≥ 1 threshold was predefined before outcome comparison because it had been used in PCa trials and pathological series [12,19,20,30,31]. It remains unvalidated in localized GG1 disease [22,36]. The exploratory ordered-category analysis showed an association across categories, but only six patients had CPS > 5 and the per-point linear model was not significant. These analyses were not intended to optimize a cutoff, and the data do not identify an optimal CPS threshold.

Upgrading in this setting denotes discordance between the sampled biopsy tissue and the RP specimen. The study cannot determine whether Gleason pattern 4 was already present at the time of biopsy but remained unsampled, and the finding should not be construed as evidence of biological progression during the interval to surgery.

Clinical relevance will depend on whether CPS improves risk assessment or decision-making beyond established clinical, imaging, and pathological variables. Current guidance permits selective use of tissue-based molecular assays but does not support routine testing for all patients with localized PCa [29]. Evidence from active-surveillance cohorts has varied for Decipher, PTEN, and the 17-gene Genomic Prostate Score [37,38,39,40]. Quantitative histological features in saturation biopsies have also been associated with adverse pathology at RP [9,41], whereas another surveillance study found that mpMRI combined with confirmatory biopsy outperformed a two-gene urinary assay for detecting clinically significant reclassification [42]. Prospective studies should evaluate whether CPS adds discrimination, calibration, and decision benefit and, importantly, whether it is associated with clinically relevant outcomes such as reclassification or progression during AS.

Because CPS combines PD-L1-positive tumor cells, lymphocytes, and macrophages, an elevated score may reflect a local inflammatory or immune-cell response rather than, or in addition to, PD-L1 expression by neoplastic cells. Although inflammatory cells associated with benign or ulcerated areas were excluded from scoring, the study did not separately quantify tumor-cell and immune-cell components, grade background chronic prostatitis, or measure inflammatory-cell density. Tumor extent in the scored block was not quantified using a standardized length or area measure, and positive-core count is an imperfect proxy for tumor burden. Residual confounding by tumor volume and inflammation cannot therefore be excluded, and the observed association may partly reflect macrophage-rich inflammation rather than intrinsic tumor aggressiveness [26]. CPS may also reflect an unsampled higher-grade focus elsewhere in the gland. Accordingly, CPS alone cannot justify reclassification of GG1 disease, exclusion from AS, or a change in treatment.

Generalizability is limited by the surgical cohort and the requirement for adequate residual biopsy tissue; men managed with AS may differ systematically from those undergoing RP. Controls were randomly sampled without stratification or matching. Non-significant descriptive *p* values and the observed standardized mean differences do not establish covariate equivalence; residual measured or unmeasured imbalance cannot be excluded despite multivariable adjustment. The 1:1 case–control design supports estimation of odds ratios but not absolute risk, predictive values, or model calibration for the full eligible cohort.

Only nine patients had an abnormal DRE, limiting the precision of estimates involving this variable. DRE was therefore omitted from the primary model and examined in conventional and Firth bias-reduced sensitivity analyses. All variance inflation factors were ≤1.06, and influence analyses did not identify any individual observation that materially altered the CPS estimate. Nevertheless, adjusted ORs are sample-dependent estimates and may be unstable when extrapolated to other populations. PSA and positive-core count were modeled as linear terms; alternative functional forms were not evaluated. No formal sample-size calculation was performed in advance. The sample size and width of the confidence intervals limit precision and permit both overestimation and underestimation of the true effect. External validation in larger prospective cohorts is required.

PD-L1 staining was performed centrally using the same SP263 antibody lot and validated positive and negative controls, while block selection, CPS scoring, and RP grading followed predefined blinding procedures. Interobserver agreement was almost perfect before consensus review. However, only one tumor-containing block from each preoperative biopsy was assessed, and heterogeneity of PD-L1 immunoreactivity across cores or tumor foci may limit the representativeness of the resulting CPS.

PD-L1 detection is influenced by glycosylation, antibody clone, staining platform, tumor context, and scoring method [22,43,44,45,46,47]. No deglycosylation was performed; the findings therefore apply to conventional SP263 immunohistochemistry under the reported fixation and staining conditions. Although deglycosylation may increase analytical detection in some settings, its clinical relevance and comparability with established assay workflows in localized PCa remain uncertain. Standardization of pre-analytical procedures and interpretation will be essential. Independent, preferably prospective, multicenter studies are required before biopsy CPS can be considered for clinical use in localized GG1 PCa.

## 4. Materials and Methods

### 4.1. Study Design and Patient Selection

This retrospective two-center case–control study included 212 consecutive men who underwent RP between January 2020 and January 2025 after a transperineal biopsy diagnosis of GG1 (GS 3 + 3) at Azienda Ospedaliera Universitaria (AOU) Policlinico “G. Martino” (Messina, Italy) or Cannizzaro Hospital (Catania, Italy). Eligibility also required at least one residual block from the biopsy closest to RP with sufficient tissue and at least 100 viable tumor cells for PD-L1 assessment. Patients were excluded if they had received neoadjuvant hormonal therapy or chemoradiotherapy before RP, undergone prior surgery for benign prostatic hyperplasia, or had incomplete records. Institutional RP reports classified 86 of 212 patients (40.6%) as GG2 and 126 as GG1; none had GG3 or higher disease. RP grade was not used as an exclusion criterion.

Each of the 126 patients classified as GG1 in the institutional RP reports was assigned a unique pseudonymous identifier. After the control pool had been finalized, 86 patients were selected by simple random sampling without replacement using the base R function sample() in R version 4.6.0 (R Foundation for Statistical Computing, Vienna, Austria). Each patient had an equal selection probability of 86/126. Sampling was completed before PD-L1 assessment and without use of clinical, biopsy-related, pathological, or institutional variables; no matching or stratification was applied. The final sample comprised 86 cases and 86 controls; 40 eligible GG1 patients were not selected.

Case–control status was assigned from institutional RP reports: all 86 patients classified as GG2 were included as cases, and blinded central review of the 172 selected RP specimens confirmed every classification. Central review of the biopsy material likewise confirmed GG1 without Gleason pattern 4 in all samples used for PD-L1 assessment. Center was included in the primary multivariable model to account for potential institutional differences. Figure 1 summarizes cohort assembly and control selection.

### 4.2. Study Endpoint

The primary outcome was pathological upgrading, defined as GG1 (GS 3 + 3) in the preoperative biopsy closest to RP and GG2 (GS 3 + 4) in the subsequent RP specimen.

### 4.3. MRI and Biopsy Protocol

The mpMRI examination performed before the biopsy selected for PD-L1 assessment was acquired on an Ingenia 1.5-T system with a 32-channel pelvic phased-array coil (Philips Healthcare, Best, The Netherlands) at AOU Policlinico “G. Martino” and on an Achieva 3.0-T system with a 16-channel phased-array pelvic coil (Philips Healthcare, Best, The Netherlands) at Cannizzaro Hospital. All examinations were interpreted according to PI-RADS v2 or v2.1 [48]. The protocol comprised multiplanar turbo spin-echo T2-weighted imaging, axial diffusion-weighted imaging, and axial dynamic contrast-enhanced imaging.

Diagnostic biopsies comprised 12–18 systematic transperineal cores plus four targeted cores for lesions scored PI-RADS ≥ 3. At AOU Policlinico “G. Martino”, targeted cores were obtained under real-time transrectal ultrasound guidance using cognitive MRI targeting with an Arietta V70a Diagnostic Ultrasound System equipped with a 7.75-MHz linear probe (Hitachi Ltd., Tokyo, Japan). At Cannizzaro Hospital, targeted cores were obtained using device-assisted MRI–ultrasound fusion with an Arietta 70 ultrasound system equipped with a biplanar transrectal probe (Hitachi Ltd., Tokyo, Japan) and Real-time Virtual Sonography (RVS; software option SOP-ARIETTA70-62) integrated as the MRI–ultrasound fusion module. For RVS-assisted targeting, MRI-defined targets were contoured and overlaid on the real-time ultrasound image before sampling. Biopsy cores were obtained using 18-gauge Tru-Cut biopsy needles with a Magnum reusable core biopsy instrument (Bard, Covington, GA, USA). At AOU Policlinico “G. Martino”, the 18-gauge needle had a 23-mm cutting length and was used with a 16-gauge coaxial access needle (BPB Medica, Mirandola, Italy). Repeat biopsies followed institutional transperineal systematic or saturation protocols, with MRI-targeted sampling when indicated, as described in published diagnostic and AS series [4,8,49,50,51].

PD-L1 was evaluated only in the biopsy closest to RP, and the biopsy-to-surgery interval was calculated in days. For that biopsy, we recorded positive-core count, PI-RADS category, biopsy type (diagnostic or repeat), DRE status (normal or abnormal), and tumor location (peripheral, anterior, or mixed peripheral and anterior). Age was recorded at initial diagnosis, whereas serum PSA was recorded at the time of the biopsy used for PD-L1 assessment and entered in all analyses. Diagnosis and grading followed International Society of Urological Pathology/World Health Organization (ISUP/WHO) criteria [5,52]. Cribriform morphology and intraductal carcinoma were recorded in both the selected biopsy and the corresponding RP specimen. Pathological stage was assigned according to the eighth edition of the Tumor-Node-Metastasis (TNM) classification [53], and RP specimens were processed as whole mounts following published protocols [54].

### 4.4. Histopathology and PD-L1 Immunohistochemistry

Histological specimens were fixed in 10% buffered formalin for 12–24 h at room temperature, embedded in paraffin, and sectioned at 4–5 μm for H&E staining. For each patient, all H&E-stained sections from the biopsy closest to RP were reviewed, and one formalin-fixed, paraffin-embedded block was selected. The chosen block contained the greatest aggregate extent of viable GG1 carcinoma, at least 100 viable tumor cells, sufficient residual tissue, and minimal crush, cautery, or sectioning artifact. Block selection was completed before PD-L1 assessment and without knowledge of RP GG. If deeper sections from the initial block were exhausted or inadequate, the next most representative tumor-containing block was used.

PD-L1 immunohistochemistry was performed centrally in seven staining runs on the Ventana Benchmark XT platform (Ventana Medical Systems, Tucson, AZ, USA) using the laboratory’s validated local SP263/OptiView protocol, the OptiView Universal DAB Detection Kit (Ventana Medical Systems, Tucson, AZ, USA), and the prediluted SP263 rabbit monoclonal antibody (Ventana Medical Systems, Tucson, AZ, USA). Each run included 22–28 study specimens. Specimens from both participating centers and both upgrading groups were distributed across runs; no run was limited to a single center or outcome group. The same prediluted SP263 antibody lot was used throughout the study. Section 4 and Section 5 μm thick were cut 1–7 days before staining to minimize antigen loss. Each run included a validated positive tissue control and a rabbit immunoglobulin G (IgG) negative reagent control, and the expected staining pattern and acceptable background were verified before interpretation. Placental tissue served as the principal positive run control, with tonsil and appendix used as additional positive-control tissues when available. Slides showing section detachment, insufficient staining, or excessive background were repeated, and only technically acceptable sections were scored. No enzymatic or chemical deglycosylation was performed before staining.

CPS was calculated as 100 times the number of PD-L1-positive tumor cells, lymphocytes, and macrophages divided by the total number of viable tumor cells. A minimum of 100 viable neoplastic cells was required, and PD-L1 staining was evaluated by light microscopy using a 20× objective on an Axio Scope.A1 microscope (Carl Zeiss Microscopy GmbH, Jena, Germany). The numerator included tumor cells showing distinct membranous staining and mononuclear inflammatory cells showing cytoplasmic and/or membranous staining within or immediately adjacent to the tumor. Neutrophils, eosinophils, plasma cells, and inflammatory cells associated with benign or ulcerated areas were excluded. Each pathologist independently calculated CPS and assigned the biopsy to the predefined category of CPS < 1 or CPS ≥ 1. The cutoff was selected before outcome comparison because it had been used in PCa trials and pathological cohorts [12,19,20,30,31], although it has not been clinically validated in localized GG1 disease [22,36]. Discordant binary classifications and differences in numerical CPS values within the same binary category were reviewed jointly using a multiheaded BX51 microscope (Olympus Corporation, Tokyo, Japan). The final consensus numerical CPS and corresponding binary category were used in all analyses. Representative staining patterns and corresponding RP grades are shown in Figure 2.

### 4.5. Pathology Review and Interobserver Agreement

Two uropathologists (A.I. and M.M.), each with more than 15 years of experience in uropathology and specific experience in PD-L1 assessment, independently reviewed the H&E and PD-L1 slides from the selected biopsies and all 172 RP specimens. Biopsy CPS was scored without knowledge of RP findings or case–control status. RP grade was assigned without knowledge of biopsy CPS or the institutional RP grade, although the reviewers knew that all patients had previously had a GG1 biopsy. Agreement before consensus review was quantified with unweighted Cohen’s κ for final RP Grade Group classification (GG1 vs. GG2) and binary biopsy CPS classification (CPS < 1 vs. CPS ≥ 1). Cohen’s κ was 0.92 for RP Grade Group and 0.83 for binary CPS. Disagreements in biopsy GG, RP GG, or CPS category were resolved by joint review, and consensus classifications were used in the analyses.

### 4.6. Ethics, Informed Consent, and Data Protection

This retrospective, non-interventional study used archived diagnostic and surgical material together with routinely collected clinical data, without additional procedures or changes in patient management. The study was conducted in accordance with the Declaration of Helsinki and approved by the Institutional Review Board of Cannizzaro Hospital (protocol 781/CS; 11 April 2019) and the Messina Ethics Committee of AOU Policlinico “G. Martino” (protocol 1611-38-21; 13 July 2021). All participants provided informed consent. Records were pseudonymized before data extraction, and no directly identifying information was retained in the research dataset.

### 4.7. Statistical Analysis

Control sampling was performed in R version 4.6.0 (R Foundation for Statistical Computing, Vienna, Austria). Statistical analyses were performed using GraphPad Prism version 10.1.0 (GraphPad Software, San Diego, CA, USA) and R version 4.6.0. Continuous variables were summarized as median [IQR], and categorical variables as *n* (%). Between-group comparisons used the Mann–Whitney U test for continuous variables and Fisher’s exact test for binary categorical variables. The four-level PI-RADS distribution was analyzed with the Fisher–Freeman–Halton exact test because of sparse cells, whereas tumor-location distributions were compared with the chi-square test. The unadjusted OR for CPS ≥ 1 versus CPS < 1 and its 95% CI were calculated from the 2 × 2 table using a log-Wald interval; the corresponding two-sided *p* value was obtained with Fisher’s exact test. Multivariable logistic regression models were fitted in R; estimates are reported with profile-likelihood 95% CIs and two-sided Wald *p* values.

The primary multivariable logistic regression model included the CPS category (≥1 vs. <1), serum PSA measured at the time of the biopsy assessed for PD-L1 (per 1 ng/mL), the positive-core count from that biopsy (per additional core), PI-RADS category for the same biopsy as an ordinal variable, and center. DRE was not included in the primary model because only nine patients had an abnormal examination. Sensitivity analyses added DRE, biopsy type, or age; omitted positive-core count; and modeled PI-RADS categorically. Because of the sparse abnormal-DRE category, an additional Firth bias-reduced logistic regression model including DRE was fitted in R [55,56]. Penalized profile-likelihood confidence intervals and likelihood-ratio *p* values were used for this model. Convergence was confirmed for all reported models.

Collinearity and model stability were examined before interpretation. Variance inflation factors (VIFs) were calculated for all predictors. Standardized mean differences (SMDs) were used as descriptive measures of case–control imbalance within the selected analytic sample and were not interpreted as evidence of equivalence. Influence was assessed using Pearson residuals, DFBETAs, and leave-one-out refitting of the CPS model. Complete data were available for all 172 patients.

CPS was additionally summarized as a continuous variable and in exploratory categories of 0, 1–5, and >5. Adjusted analyses evaluated an ordered-category association across these categories and CPS per one-point increase; the ordered model imposed a common log-odds increment and was not interpreted as demonstrating a dose–response. These analyses were not used to optimize a cutoff. PSA and positive-core count were modeled as linear terms. The sample size was determined by the inclusion of all 86 eligible cases and an equal number of randomly selected controls. All tests were two-sided, with *p* < 0.05 denoting statistical significance. The 1:1 case–control sampling design allowed estimation of ORs but not absolute risks or predictive values for the 212 eligible patients and was not used to assess model calibration. Table 1 comparisons were treated as exploratory descriptions; no multiplicity correction was applied, and no inferential conclusion was based on those comparisons.

## 5. Conclusions

In this two-center case–control study, biopsy PD-L1 CPS ≥ 1 was associated with upgrading from GG1 on biopsy to GG2 at RP after adjustment for clinical, biopsy, imaging, and center covariates. The association was consistent in Firth bias-reduced and alternative-specification sensitivity analyses. This study does not establish an optimal CPS threshold, predictive performance, or clinical utility, and CPS should not be used in isolation to determine eligibility for AS or treatment. Independent prospective multicenter studies using standardized pre-analytical procedures, staining protocols, and scoring criteria, with outcomes such as reclassification or progression during AS, are needed to determine whether CPS provides clinically useful information beyond an established clinicopathological and imaging assessment.

## Figures and Tables

**Table 1:** Baseline clinicopathological characteristics according to upgrading status (*N* = 172).

Variable	Not Upgraded at RP (GG1; GS 3 + 3, *n* = 86)	Upgraded at RP (GG2; GS 3 + 4, *n* = 86)	*p* Value
Center, *n* (%)	Messina: 48 (55.8); Catania: 38 (44.2)	Messina: 52 (60.5); Catania: 34 (39.5)	0.643
Biopsy type, *n* (%)	Diagnostic: 39 (45.3); Repeat: 47 (54.7)	Diagnostic: 36 (41.9); Repeat: 50 (58.1)	0.759
Age at diagnosis, years, median [IQR]	65.75 [62.30–68.95]	64.60 [61.10–68.30]	0.223
PSA at biopsy, ng/mL, median [IQR]	8.56 [6.71–10.64]	8.34 [6.97–10.88]	0.982
Abnormal DRE at biopsy, *n*/*N* (%)	2/86 (2.3)	7/86 (8.1)	0.168
Positive cores in biopsy, median [IQR]	6 [4–7]	6 [5–8]	0.376
PI-RADS category at biopsy, *n* (%)	2: 17 (19.8); 3: 36 (41.9); 4: 30 (34.9); 5: 3 (3.5)	2: 23 (26.7); 3: 34 (39.5); 4: 26 (30.2); 5: 3 (3.5)	0.733
Tumor location in biopsy, *n* (%)	Peripheral: 58 (67.4); Anterior: 18 (20.9); Mixed peripheral and anterior: 10 (11.6)	Peripheral: 62 (72.1); Anterior: 18 (20.9); Mixed peripheral and anterior: 6 (7.0)	0.567

Abbreviations: *n*, number of patients; *N*, total number of patients; GS, Gleason score; IQR, interquartile range; PSA, prostate-specific antigen; DRE, digital rectal examination; PI-RADS, Prostate Imaging Reporting and Data System; RP, radical prostatectomy; GG, grade group. The *p* values are exploratory descriptive comparisons; no multiplicity correction was applied, and they should not be interpreted as tests establishing baseline equivalence.

**Table 2:** Unadjusted association between biopsy CPS ≥ 1 and pathological upgrading at RP (*N* = 172).

PD-L1 CPS Category	Not Upgraded, *n* (%)	Upgraded, *n* (%)	Unadjusted OR (95% CI)	*p* Value
CPS < 1	68 (79.1)	50 (58.1)	Reference	—
CPS ≥ 1	18 (20.9)	36 (41.9)	2.72 (1.39–5.33)	0.005

Abbreviations: *n*, number of patients; *N*, total number of patients; PD-L1, programmed death-ligand 1; CPS, combined positive score; OR, odds ratio; CI, confidence interval; RP, radical prostatectomy. Percentages were calculated within the non-upgraded and upgraded groups. The OR and log-Wald 95% CI compare CPS ≥ 1 with CPS < 1; the *p* value was obtained from a two-sided Fisher’s exact test.

**Table 3:** Primary multivariable logistic regression analysis of pathological upgrading at RP (*N* = 172).

Variable	Adjusted OR	95% CI	*p* Value
PD-L1 CPS ≥ 1 vs. <1	2.91	1.47–5.97	0.003
PSA at biopsy, per 1 ng/mL	1.01	0.91–1.11	0.888
Positive cores in biopsy, per additional core	1.04	0.89–1.22	0.626
PI-RADS category at biopsy, per one-category increase	0.75	0.50–1.10	0.146
Center, Messina vs. Catania	1.29	0.68–2.47	0.432

Abbreviations: *N*, total number of patients; OR, odds ratio; CI, confidence interval; CPS, combined positive score; PD-L1, programmed death-ligand 1; PSA, prostate-specific antigen; PI-RADS, Prostate Imaging Reporting and Data System; RP, radical prostatectomy. PI-RADS was modeled as an ordinal variable. Confidence intervals are profile-likelihood intervals; *p* values are two-sided Wald tests.

## Data Availability

Supplementary Tables S1–S4 are provided with this article. The pseudonymized dataset for the 172 study participants is available from the corresponding author upon reasonable request, subject to institutional, ethical, and data-protection requirements.

## Supplementary Materials

The following supporting information can be downloaded at https://www.mdpi.com/article/10.3390/ijms27177878/s1.
