# Supplementary material for: Association Between Biopsy PD-L1 Combined Positive Score and Pathological Upgrading at Radical Prostatectomy in Grade Group 1 Prostate Cancer: A Retrospective Study
Source: Int J Mol Sci. 2026 Sep 3;27(17):7878. doi: 10.3390/ijms27177878 (PMC13566208; doi:10.3390/ijms27177878)
Supplement: Supplementary file 1 [file ijms-27-07878-s001.zip › ijms-4503810-supplementary.pdf]

Table S1. Distribution and exploratory modeling of biopsy CPS

Panel A. Distribution of CPS by pathological upgrading status.

CPS summary	Not upgraded (n = 86)	Upgraded (n = 86)
Median [IQR]	0 [0–0]	0 [0–1]
Range	0–20	0–20
CPS = 0, n (%)	68 (79.1)	50 (58.1)
CPS 1–5, n (%)	16 (18.6)	32 (37.2)
CPS > 5, n (%)	2 (2.3)	4 (4.7)

Panel B. Exploratory adjusted analyses of CPS.

CPS specification	Adjusted OR	95% CI	p value
Ordered categories (0, 1–5, >5), per category increase	2.44	1.35–4.62	0.004
Continuous CPS, per one-point increase	1.09	0.97–1.27	0.208

Models were adjusted for PSA at the selected biopsy, positive-core count, PI-RADS category, and center. Continuous and category-based analyses used the final numerical CPS agreed by consensus. The ordered-category model treated 0, 1–5, and >5 as ordered levels and imposed a common log-odds increment across transitions. These analyses were exploratory and were not used to select or optimize a cutoff. Only six patients had CPS > 5. OR, odds ratio; CI, confidence interval; CPS, combined positive score; IQR, interquartile range.

Table S2. Sensitivity analyses for the association between CPS ≥ 1 and upgrading

Analysis	Adjusted OR	95% CI	p value
Primary conventional model: PSA, positive cores, ordinal PI-RADS, center	2.91	1.47–5.97	0.003
Primary model + DRE	2.91	1.46–5.99	0.003
Firth bias-reduced model including DRE	2.77	1.41–5.61	0.003
Primary model + biopsy type	3.14	1.56–6.58	0.002
Primary model + age	2.94	1.48–6.04	0.003
Primary model without positive-core count	2.98	1.51–6.08	0.002
PI-RADS modeled categorically	2.89	1.45–5.95	0.003

All estimates compare CPS ≥ 1 with CPS < 1. Conventional models report profile-likelihood 95% CIs and two-sided Wald p values; the Firth model reports a penalized profile-likelihood 95% CI and a penalized likelihood-ratio p value. DRE, digital rectal examination; PI-RADS, Prostate Imaging Reporting and Data System.

Table S3. Collinearity and influence diagnostics

Panel A. Variance inflation factors in the model containing CPS, PSA, positive-core count, ordinal PI-RADS, DRE, and center.

Predictor	VIF
PD-L1 CPS category	1.04
PSA	1.02
Positive-core count	1.06
PI-RADS category	1.06
Abnormal DRE	1.04
Center	1.04

Panel B. Influence diagnostics for the primary conventional logistic-regression model.

Diagnostic	Result
Maximum absolute Pearson residual	1.61
Observations with |Pearson residual| > 2	0
Absolute DFBETA screening threshold (2/√172)	0.153
Maximum absolute DFBETA for the CPS coefficient	0.225
Observations above the CPS DFBETA screening threshold	8
Leave-one-out CPS OR range	2.80–3.16
Largest leave-one-out p value	0.0041
Leave-one-out refits with p < 0.05	172/172

A VIF >5 was considered potentially concerning. The DFBETA threshold was used as a screening value, not as an automatic deletion rule. No observation was removed. Leave-one-out analyses re-estimated the complete primary model after omitting one patient at a time. VIF, variance inflation factor; OR, odds ratio; CPS, combined positive score.

Table S4. Standardized mean differences (SMDs) in the primary analytic sample

Covariate	Signed SMD (upgraded minus non-upgraded)
Age	−0.183
PSA	−0.025
Positive-core count	0.156
PI-RADS (ordinal)	−0.141
Abnormal DRE	0.263
Messina center	0.094
Repeat biopsy	0.070
Anterior tumor location	0.000
Mixed tumor location	−0.161

SMD methods: Signed SMDs are reported as upgraded minus non-upgraded. Continuous-variable SMDs used the difference in means divided by the pooled standard deviation; binary variables used the difference in proportions divided by the pooled binomial standard deviation. These descriptive measures characterize covariate imbalance within the selected analytic sample and do not establish equivalence.
